# Evaluation of pathogen from the FilmArray meningitis/encephalitis panel and recommendations on atypical findings

**DOI:** 10.1055/s-0044-1779035

**Published:** 2024-02-05

**Authors:** Luiz Gustavo Ferreira Côrtes, Mariana Menezes Maldonado, Paula Celia Mariko Koga, Kelly Aline de Souza Santiago, Gustavo Bruniera Peres Fernandes, Maira Marranghello Maluf, Marinês Dalla Valle Martino

**Affiliations:** 1 Hospital Israelita Albert Einstein, Laboratório Clínico, São Paulo SP, Brazil.

**Keywords:** Meningoencephalitis, Inflammation, Microbiology, Meningoencefalite, Inflamação, Microbiologia

## Abstract

**Background** Infectious meningoencephalitis is a potentially fatal clinical condition that causes inflammation of the central nervous system secondary to the installation of different microorganisms. The FilmArray meningitis/encephalitis panel allows the simultaneous detection of 14 pathogens with results in about one hour.

**Objective** This study is based on retrospectively evaluating the implementation of the FilmArray meningitis/encephalitis panel in a hospital environment, highlighting the general results and, especially, analyzing the consistency of the test results against the clinical and laboratory conditions of the patients.

**Methods** Data were collected through the results reported by the BioFire FilmArray system software from the meningitis/encephalitis panel. The correlated laboratory tests used in our analysis, when available, included biochemical, cytological, direct and indirect microbiological tests.

**Results** In the analyzed period, there were 496 samples with released results. Of the total of 496 samples analyzed, 88 (17.75%) were considered positive, and 90 pathogens were detected, and in 2 of these (2.27%) there was co-detection of pathogens. Viruses were the agents most frequently found within the total number of pathogens detected. Of the 496 proven samples, 20 (4.03%) were repeated, 5 of which were repeated due to invalid results, 6 due to the detection of multiple pathogens and 9 due to disagreement between the panel results and the other laboratory tests and/or divergence of the clinical-epidemiological picture. Of these 20 repeated samples, only 4 of them (20%) maintained the original result after repeating the test, with 16 (80%) being non-reproducible. The main factor related to the disagreement of these 16 samples during retesting was the detection of bacterial agents without any relationship with other laboratory tests or with the patients' clinical condition.

**Conclusion** In our study, simply reproducing tests with atypical results from the FilmArray meningitis/encephalitis panel proved, in most cases, effective and sufficient for interpreting these results.

## Resumo

**Antecedentes** A meningoencefalite infecciosa é uma condição clínica potencialmente fatal que causa inflamação do sistema nervoso central secundária à instalação de diversos microrganismos. O painel de meningite/encefalite FilmArray permite a detecção simultânea de 14 patógenos, com resultados em cerca de uma hora.

**Objetivo** Este estudo baseia-se em avaliar retrospectivamente a implementação do painel de meningite/encefalite FilmArray em ambiente hospitalar, destacando os resultados gerais e, principalmente, analisando a consistência dos resultados do teste frente às condições clínicas e laboratoriais dos pacientes.

**Métodos** Os dados foram coletados por meio dos resultados relatados pelo software do sistema BioFire FilmArray do painel de meningite/encefalite. Os exames laboratoriais correlacionados utilizados em nossa análise, quando disponíveis, incluíram exames bioquímicos, citológicos, microbiológicos diretos e indiretos.

**Resultados** No período analisado, foram 496 amostras com resultados divulgados. Do total de 496 amostras analisadas, 88 (17,75%) foram consideradas positivas e 90 patógenos foram detectados, sendo que em duas destas (2,27%) houve codetecção de patógenos. Os vírus foram os agentes mais frequentemente encontrados dentro do total de patógenos detectados. Das 496 amostras analisadas, 20 (4,03%) foram repetidas, sendo 5 repetidas por resultado inválido, 6 pela detecção de múltiplos patógenos e 9 por discordância dos resultados do painel com os demais exames laboratoriais e/ou divergência do quadro clínico-epidemiológico. Destas 20 amostras repetidas, apenas 4 delas (20%) mantiveram o resultado original após a repetição do teste, sendo 16 (80%) não reprodutíveis. O principal fator relacionado à discordância destas 16 amostras na retestagem foi a detecção de agentes bacterianos sem qualquer relação com os demais exames laboratoriais ou com o quadro clínico dos pacientes.

**Conclusão** Em nosso estudo, a simples repetição dos testes com resultados atípicos do painel de meningite/encefalite FilmArray mostrou-se, na maior dos casos, efetiva e suficiente para a interpretação destes achados.

## INTRODUCTION

Infectious meningoencephalitis (ME) is a serious and potentially fatal clinical condition that causes inflammation of the central nervous system secondary to the installation of different microorganisms. As it is associated with significant morbidity and mortality, especially in cases of bacterial meningitis, prompt diagnosis and treatment are particularly critical. 1 2 Furthermore, the costs associated with these infections are considerable, both in the short term, linked to hospitalization and treatment costs, and in the long term, considering the possible productive losses of the affected individuals. 3

Testing of cerebrospinal fluid (CSF) often includes Gram staining, culture, immunological, and molecular methods, in addition to biochemical and cellular analysis. These methods present varying sensitivity and specificity and may take several days and require a large sample volume for complete analysis. While microbiological culture is considered the gold standard for the diagnosis of bacterial and fungal infections, nucleic acid tests are routinely used in the diagnosis of viral infections. 4 However, it is estimated that up to 50% of cases of encephalitis and 60% of cases of meningitis have an unknown etiology. 1

In recent years, the FDA (US Food and Drug Administration) has approved molecular panels for the simultaneous detection of multiple pathogens in respiratory, gastrointestinal, meningoencephalic, and, more recently, bloodstream and pulmonary infections, simplifying the workflow in the laboratory, streamlining delivering results, helping to limit antibiotic therapy and hospitalization costs, and improving clinical practice. 5 6

The FilmArray meningitis/encephalitis panel (FilmArrray Meningitis/Encephalitis panel, BioFire Diagnostics, LLC, Salt Lake City, Utah) is a multiplex molecular nested PCR (polymerase chain reaction) panel with fully automated melting curve analysis, approved by the FDA in 2015. The test allows the simultaneous detection of 14 pathogens (Escherichia coli K1, H. influenzae, Listeria monocytogenes, N. meningitidis, Streptococcus agalactiae, S. pneumoniae, cytomegalovirus [CMV], enterovirus [EV], herpes simplex virus 1 [HSV-1], herpes simplex virus 2 [HSV-2], human herpesvirus 6 [HHV-6], human parechovirus [HPeV], varicella-zoster virus [VZV], and Cryptococcus neoformans/C. gattii) from 200µL of cerebrospinal fluid sample, with results available in approximately one hour. 7

Since the panel was approved, several studies have evaluated its performance and a few have evaluated its clinical application. However, as its use has expanded, more data are needed to evaluate its appropriate use and interpret the results. Thus, the current study is based on retrospectively evaluating the implementation of the test in a hospital environment, highlighting the general results and, especially, analyzing the consistency of the test results against the clinical and laboratory conditions of the patients. Finally, we aimed to critically analyze the test, to provide recommendations regarding its best use.

## METHODS

Data were collected from the results reported by the BioFire FilmArray system software from the meningitis/encephalitis panel. The tests were performed at the Clinical Laboratory of the Hospital Israelita Albert Einstein, Morumbi Unit, according to the manufacturer's instructions, in the period from April 2018 to May 2021, from patients hospitalized or seen in the emergency room. In addition, data were retrieved on the results of other laboratory tests and on the clinical condition of the patients when available in the laboratory computer system. Outpatients were excluded and there was no age restriction in our analysis. Samples from the same patient analyzed at different times were not excluded, although this occurred in only two patients.

The correlated laboratory tests used in our analysis, when available, included biochemical (lactate, protein, and glucose dosage in mg/dL), cytological (total number of cells and differential leukocyte count p/mm3), direct microbiological (Gram staining), and indirect tests (culture and identification). It is important to point out that, as this is a retrospective observational study, not all samples contained all the information.

Based on the findings of this study, we carried out an integrated analysis of the data and a review and search in the literature on the results and experience of other centers that perform the test, especially regarding the strengths and limitations of the test.

As this retrospective review presented a minimal risk to participants, a waiver of consent was granted by the Research Ethics Committee of the Hospital Israelita Albert Einstein (CAAE: 63977522.0.0000.0071).

The median was calculated for all continuous variables using JAMOVI software, version 1.6 (The Jamovi Project, Sydney, Australia). 8

## RESULTS

In the analyzed period, 516 tests were carried out, 20 of which were repeated, resulting in a total of 496 samples with released results. Of the total number of samples, 257 (51.81%) were from male patients and 239 (48.19%) from female patients. Patient ages ranged from 2 weeks to 95 years, with 168 (33.87%) patients between 0 and 17 years, 203 (40.93%) patients between 18 and 59 years, and 125 (25.20%) patients aged over 60 years.

Of the total of 496 samples analyzed, 88 (17.75%) were considered positive and released after a final evaluation carried out by an analyst together with a medical professional from the clinical laboratory, in correlation with the other laboratory tests and clinical-epidemiological picture, when available.

The 88 positive samples released detected a total of 90 pathogens, and in 2 of these (2.27%) there was co-detection of pathogens (HHV-6 + EV and S. agalactiae + EV). The distribution of the 90 pathogens according to category was: 80 viruses (88.89% of the detected pathogens or 16.13% of the total samples tested), 7 bacteria (7.78% of the detected pathogens or 1.41% of the total samples tested), and 3 fungi (3.33% of the detected pathogens and 0.60% of the total tested samples), as detailed and specified in Table 1 .

**Table 1:** Distribution of agents detected in positive samples of cerebrospinal fluid through the syndromic molecular panel

Microorganism	Positive samples (n)	Percentage among positive samples
*Escherichia coli* K1	0	0%
*Haemophilus influenzae*	1	1.1%
*Listeria monocytogenes*	0	0%
*Neisseria meningitidis*	1	1.1%
*Streptococcus agalactiae*	1	1.1%
*Streptococcus pneumoniae*	4	4.5%
Cytomegalovirus	2	2.2%
Enterovirus	48	53.4%
Herpes simplex virus 1	2	2.2%
Herpes simplex virus 2	9	10%
Human herpesvirus 6	3	3.3%
Human parechovirus	0	0%
Varicella zoster virus	16	17.8%
*Cryptococcus neoformans/gatti*	3	3.3%
Total	90	100%

As shown in Table 1 , viruses were the agents most frequently found within the total number of pathogens detected, with Enterovirus (53.4%) being the most prevalent, followed by the varicella-zoster virus. Regarding bacteria, the highest prevalence was Streptococcus pneumoniae (4.5%).

The medians of the lactate, protein, and glucose dosage results, the total leukocyte count, and the percentage of polymorphonuclear cells in the differential count are detailed in Table 2 , according to the group of pathogens.

**Table 2:** Median values for lactate, protein and glucose dosage, and leukocyte count (global and differential polymorphonuclear cells) in positive cases, by group of pathogens

	Lactate (mg/dL)	Protein (mg/dL)	Glucose (mg/dL)	Leukocytes (p/mm3)	PMN (%)
Virus	20.9 (17.15-24.65)	55 (35-89)	56 (47-65)	207 (51-354)	14 (3-47)
Bacteria	69.0 (21.5-88.6)	121 (55-258)	42 (38-59)	375 (230-3072)	74 (36-85)
Fungus	51.85 (47.7–)	141 (50–)	35 (25–)	151 (13–)	73 ^†^ (73-73)

Note: ^†^ only one patient had a differential leukocyte count.

Of the 496 samples processed, 20 were repeated, resulting in a repetition rate of 4.03%, with 5 samples repeated due to invalid results, 6 due to the detection of multiple pathogens, and 9 due to disagreement between the panel results and other laboratory tests and/or divergence from the clinical-epidemiological picture.

The 5 samples that were repeated due to invalid results showed a valid and conclusive result on repeat (with or without pathogen detection). Of the 6 samples that were repeated because they presented more than one pathogen, as recommended by the test manufacturer, 5 presented either only one pathogen or none on repeat, with only one reproduced sample (detecting the same 2 pathogens as the first test). Of the other 9 samples repeated due to disagreement with other laboratory tests and/or the clinical-epidemiological picture, 6 presented different results in the repetition, with no pathogen detected. These 6 cases presented results of biochemical and cytological exams without or with only slight alterations, in addition to negative microbiological tests. The other 3 samples maintained the same results after repeating the test.

**Figure 1:**
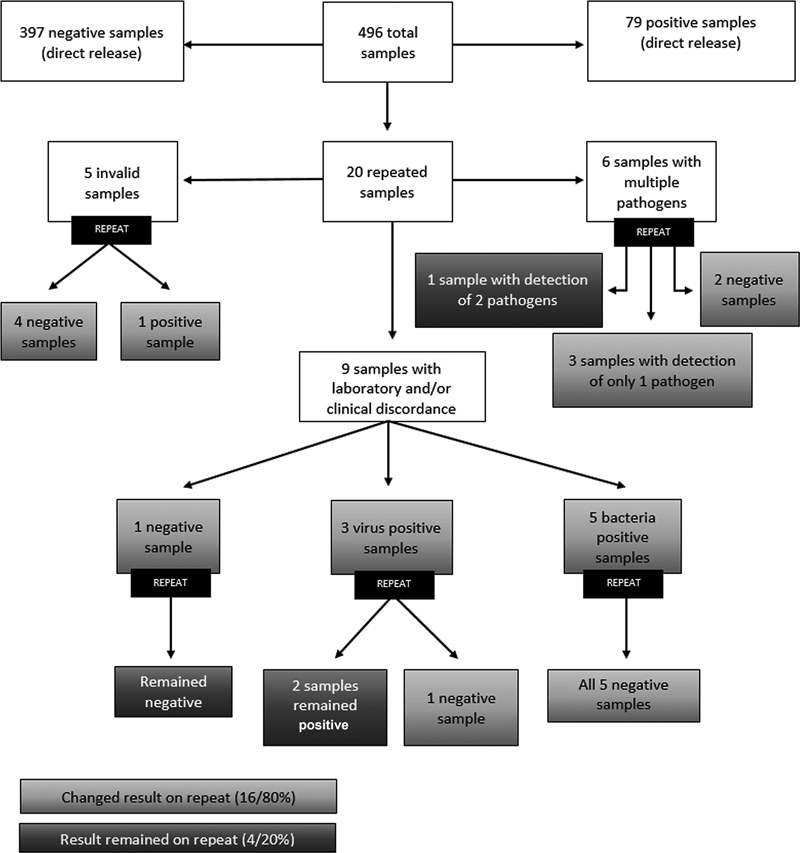
Summary of the results of the tested samples, with details of repeated cases.

Figure 1 summarizes the results of the samples tested, with details of the repeated cases. Chart 1 presents an overview of all repeated cases, with details of the results of other related laboratory tests, as well as a breakdown of the pathogens identified in the initial and repeated tests and, finally, the pathogen considered in releasing the final result after data integration analysis. The notes contain the possible reasons for the disagreement between the repeated tests and the criteria used in the final release.

**Chart 1.** Overview of all repeated cases.

Sex / Age	Lactate (mg/dL)	Protein (mg/dL)	Glucose (mg/dL)	Total Leukocytes p/mm3 (differential of PMN in %)	Microbiological test (culture)	First test	Second test	Final release
M 18	20.8	46	51	277 (2%)	Without growth	Invalid	Undetectable	Undetectable
M 42	11.1	28	51	23 (9%)	Without growth	Invalid	Undetectable	Undetectable
M 4	24.5	63	64	1115 (87%)	Without growth	Invalid	Undetectable	Undetectable
M 4	17	34	50	250 (66%)	Without growth	Invalid	Undetectable	Undetectable
M 71	69	141	42	530 (74%)	Without growth	Invalid	*Haemophilus influenzae*	*Haemophilus* *influenzae*
F 31	18.8	31	47	168 (3%)	Without growth	*Haemophilus influenzae* , Enterovirus	Enterovirus	Enterovirus ^a^
M 11 m	14.4	67	43	14 (12%)	Without growth	*Listeria monocytogenes* , HSV 2	Undetectable	Undetectable ^a^
M 3	18	101	39	48 (12%)	Without growth	*Streptococcus pneumoniae* , HHV6	Undetectable	*Streptococcus pneumoniae* ^bc^
M 28	26.6	64	62	85 (12%)	Without growth	VZV, HHV6	VZV	VZV ^c^
M 35	50	128	98	182 (NR)	Without growth	VZV, CMV	VZV	VZV ^c^
M 9	21.5	55	59	230 (33%)	Without growth	*Streptococcus agalactiae* , Enterovirus	*Streptococcus agalactiae* , Enterovirus	Streptococcus agalactiae, Enterovirus
F 48	13.6	29	55	1 (NR)	Without growth	*Streptococcus agalactiae*	Undetectable	Undetectable ^a^
M 39	19.5	40	59	94 (51%)	Without growth	Enterovirus	Enterovirus	Enterovirus
M 47	15	25	57	3 (NR)	Without growth	*Haemophilus influenzae*	Undetectable	Undetectable ^a^
F 5	31.9	14	65	1 (NR)	Without growth	*Haemophilus influenzae*	Undetectable	Undetectable ^a^
M 32	13.7	68	56	150 (0%)	Without growth	HHV6	Undetectable	Undetectable ^c^
F 69	25.7	62	69	250 (1%)	Without growth	HSV2	HSV2	HSV2
F 15 m	15.3	24	61	55 (72%)	Without growth	*Escherichia coli* K1	Undetectable	Undetectable ^a^
F 7 m	12.8	20	59	7 (5%)	Without growth	*Streptococcus agalactiae*	Undetectable	Undetectable ^a^
M 16 m	15.8	54	58	120 (40%)	Without growth	Undetectable	Undetectable	Undetectable

Abbreviations: m, months; NR, Unrealized.

Notes: ^a^ Possible contamination (other laboratory tests without significant changes and/or incompatible clinical-epidemiological picture); ^b^ Possible low bacterial load (patient in treatment control with previous positive panel for S. pneumoniae in the previous week); ^c^ Possible latent virus.

**Table:** 

Invalid cases
Cases of detection of multiple pathogens
Cases of discordance with other tests and/or clinical-epidemiological picture
Discordant cases on repeat testing

## DISCUSSION

Cases of meningoencephalitis commonly present with similar symptoms and the causal agents involved cannot be clinically differentiated, imposing a diagnostic challenge. 9 Routine tests available today, such as Gram staining, culture, antigen detection, biochemical tests, and cell counts, are time-consuming and lack sensitivity and specificity. In addition, the available sample volume may not be sufficient if several tests are performed, and prior administration of antibiotics may alter the results of microbiological examinations. Molecular testing of nucleic acid has overcome part of these difficulties but demands specialized labor and more technological equipment. The FilmArray meningitis/encephalitis molecular panel was proposed to simplify the workflow in the laboratory.

Although studies are available using data from the FilmArray meningitis/encephalitis panel results, comparisons between them are limited by sample selection criteria, the diversity of study designs, and the profile of each laboratory.

Our investigation showed a total positivity rate of 17.75%, represented mostly by viral agents (16.13% of the total samples tested or 88.89% of the positive samples) and to a lesser extent by bacterial agents (1.41% of the total samples tested or 7.78% of the positive samples). Other studies showed a more dispersed detection rate, ranging from 5.7% to 40% for viruses and 1.41% to 17.54% for bacteria, justified mainly by the heterogeneity of the population tested. 10 11 12 13 14 15 16 17 In one study, for example, testing was performed only on samples with a high pre-test probability, when the microbiological or cytological exams were altered or the clinical condition justified the test. 17

The prevalence of pathogens found in our case series was similar to that in the majority of other studies, with Enterovirus being the most detected virus and S. pneumoniae being the most prevalent bacteria. An exception was found in a European series, in which S. agalactiae was the most commonly detected bacterial agent. 15 In addition, we found the second highest prevalence among viruses for the varicella-zoster virus, which differs from some other studies that showed HHV-6 as the second most prevalent. One can consider, however, the possibility that the detection of HHV-6 had no clinical relevance, since it is a virus whose latency is common, as shown by a study in New York, in which 26.6% of positive samples were considered clinically insignificant, with almost all of them being represented by HHV-6. 13 If it were actually considered positive and clinically relevant, its prevalence would have surpassed that of the Enterovirus in the mentioned study.

In our study, the low reproducibility of the assay after repeating the tests with atypical results is quite striking. We found 20 repeat samples, among which, except for the 5 that were retested after a first invalid result, almost 3/4 (11/15 or 73.33%) showed different results in the repeat (highlighted in red in Chart 1 ). The possible causes for this non-reproducibility were: the detection of a latent viral agent such as HHV-6 or CMV (4 cases), possible low pathogen load in the sample (1 case), or sample contamination (7 cases).

The main disagreement in the retesting of the samples was the detection of bacterial agents without any relationship with the other laboratory tests, including microbiological tests, or with the clinical and epidemiological status of the patients. If the results of the first test were accepted and released indiscriminately, without critical analysis considering the other laboratory tests or the clinical and epidemiological status of the patients, there would have been 7 more positive cases for bacteria, which would double the number of cases that were effectively released (the positivity for bacteria in our series from 1.41% to 2.82%). Thus, the simple practice of repeating discordant tests proved to be quite effective in resolving positive (false) cases.

The multicenter study that supported the FDA's approval of the test showed a considerable rate of false-positive samples. 10 In that study, involving 1560 specimens, 9 samples out of a total of 22 positive for bacterial agents, from 5 different centers, did not have any correlation with the other laboratory tests (41% false positives). The low rate of positivity for bacteria in the study was also the subject of criticism from other authors, who raised doubts about the accuracy of the test for these targets and requested a robust assessment of this panel component. 1 5 18 19 20

In a meta-analysis, the total percentage of false-positive cases was 11.4% (92 pathogens out of a total of 807 detected), with the highest proportion of cases being Streptococcus pneumoniae, followed by Streptococcus agalactiae. 21 However, after judging the test results in conjunction with clinical information and/or after checking with other laboratory tests, the percentage of false-positive samples dropped to 4%, making clear the importance of reviewing the results and the relevance and frequency of false-positive samples.

Our study included a population of patients with a profile similar to that of the reference study that was the basis for FDA approval and showed a similar prevalence of agents in the group of viruses and bacteria. Our rate of false positivity for bacteria was, however, even higher. Although we carried out the joint analysis of the results, substantially reducing the false-positive cases, we still included one sample released (patient “M 9” from Chart 1 ) that proved to be false-positive for the bacterial agent, even though the repeated test was consistent with the initial test and detected the same pathogens. It is possible that contamination by the bacterial agent occurred during the collection of the test or prior to handling of the sample in the laboratory.

Based on the observations of the reference study and the serious consequences of a false-positive result, the manufacturer itself recommended the adoption of a strict procedure in the laboratory in order to minimize contamination in the testing environment. 10 Furthermore, to mitigate these possibilities of incorrect or inconclusive results, based on our findings and the experience of other studies, we created a panel with recommendations to be taken into account when using and interpreting the test ( Chart 2 ).

**Chart 2:** Recommendations to be taken into account when using and interpreting the test

- Carrying out rigorous antisepsis of the lumbar puncture site;
- Use of personal protective equipment (including mask and goggles), in addition to sterile flasks and fields at the time of CSF collection;
- Rigid monitoring of laboratory procedures to avoid contamination of samples in handling, transport and storage;
- Use of a security cabin dedicated exclusively to molecular tests;
- Joint analysis of the results with other laboratory tests, including using the repetition of the test or the use of parallel conventional molecular methods to confirm any atypical findings or even rigorously in all positive cases (especially in cases of detection of a bacterial agent);
- Verification of consistency of results with clinical data and diagnostic hypothesis;
- Evaluation of the possibility of latency of the detected pathogen, unrelated to the current clinical picture;
- Assessment of sociodemographic and epidemiological characteristics of patients, as well as seasonality, risk factors and immune status;
- Research on the possibility of previous or ongoing treatment, with the use of antimicrobials before sample collection;
- Verification of melting curves and, if they are atypical, repeat the test or consider alternative tests*

Note: *The interest in verifying melting curves should be further investigated in further studies. 15

Additionally, other conditions need to be considered when interpreting the results, for example, the possibility of infection by other pathogens not included in the panel, such as West Nile Virus, Mycobacterium tuberculosis, Borrelia burgdorferi, and Histoplasma capsulatum. In a study of 42 patients evaluating FilmArray panel results in Taiwan, 5 negative samples tested positive using other tests for locally prevalent pathogens (Japanese Encephalitis Virus, Adenovirus, *Leptospira sp.* , and *T. pallidum* ). 14 In addition, non-community-acquired infections should be considered when relevant, such as those associated with shunts or postoperative infections, in which germs such as *Staphylococcus spp.* , *Cutibacterium acnes, Candida spp., Pseudomonas spp., Acinetobacter spp.* , and other enteric Gram-negative bacilli (with the exception of E. coli K1) may be present.

Furthermore, the fact that the result is positive for a pathogen should not rule out the possibility that this result is a false positive and that there is another microorganism in the sample that is not included in the panel. In one case description, panel detection of HSV-1 delayed the diagnosis of tuberculous meningitis in an immunocompromised patient, leaving severe neurologic sequelae. 18 On that occasion, when the test was repeated on the remaining sample, the panel result was negative and another conventional singleplex PCR test performed separately for HSV-1 was also negative, unlike the PCR test for *M tuberculosis* which gave a positive result.

It is also important to point out that viruses such as HHV-6 and CMV do not usually affect immunocompetent individuals, and can be detected in latency. Since the panel is not capable of distinguishing between latent or active infection (primary or secondary due to reactivation), the analysis of positive results for one of these viruses must be carried out in a judicious manner, in addition to considering other data. The mere presence of the viral agent does not guarantee infection, as indicated in one of the warning messages of the FilmArray® system. In these cases, repeating the test may not detect the latent viral agent, most likely due to the low viral load, as observed in our case series.

This study has limitations that need to be discussed. The impact of the panel in terms of cost-effectiveness, as well as the impact of the cost of confirming or supplementing inconsistent or false-positive cases with other tests was not analyzed. Despite this, economic analysis models showed that syndromic testing in both adults and children with suspected ME was not more expensive than standard care and, most importantly, that testing cases with only abnormal CSF was not cost-effective. 22 23 24 25 Our sample pool came from a private laboratory and, therefore, information is lacking for the discussion of access and implementation in the public system. Furthermore, false-negative results were not evaluated, as we did not have comparison tests for all viral targets and did not have access to clinical data from all patients included in the study, only from those with positive panel results.

In conclusion, our results corroborate other studies in confirming the usefulness of the FilmArray meningoencephalitis panel in the diagnosis of central nervous system infections when combined (and not opposed) with other laboratory tests and in conjunction with clinical data. Despite the enthusiasm for using this simplified and miniaturized diagnostic platform, which does not require an operator with skills in molecular biology in the analytical phase, we were able to show the need for its use with caution, especially in the special situations reported.

The use of the test by professionals who are familiar with its characteristics, limitations, and performance, particularly due to the possibility of false-positive results, is crucial for better diagnostic accuracy, correct antimicrobial administration, and a reduction in hospital costs. The simple repetition of the test for cases of detection of multiple pathogens or discrepancies with other laboratory or clinical data was, in most cases, sufficient and elucidative for the final interpretation of the result.
